# A verified habitat suitability model for the intertidal rock oyster, *Saccostrea cucullata*

**DOI:** 10.1371/journal.pone.0217688

**Published:** 2019-06-11

**Authors:** Mohammed Shah Nawaz Chowdhury, Johannes W. M. Wijsman, M. Shahadat Hossain, Tom Ysebaert, Aad C. Smaal

**Affiliations:** 1 Institute of Marine Sciences, University of Chittagong, Chittagong, Bangladesh; 2 Wageningen Marine Research, Wageningen University and Research, Yerseke, the Netherlands; 3 Aquaculture and Fisheries Group, Wageningen University and Research, Wageningen, the Netherlands; 4 NIOZ Yerseke, Royal Netherlands Institute for Sea Research, Department of Estuarine and Delta Systems, and Utrecht University, Yerseke, the Netherlands; University of North Carolina at Chapel Hill, UNITED STATES

## Abstract

There is growing interest to restore oyster populations and develop oyster reefs for their role in ecosystem health and delivery of ecosystem services. Successful and sustainable oyster restoration efforts largely depend on the availability and selection of suitable sites that can support long-term growth and survival of oysters. Hence, in the present study a habitat suitability index (HSI) model was developed for the intertidal rock oyster (*Saccostrea cucullata*), with special attention: (1) to the role of the monsoon in the suitability of oyster habitats, and (2) to identify potential suitable sites along the south-eastern Bangladesh coast. Seven habitat factors were used as input variables for the HSI model: (1) water temperature; (2) salinity; (3) dissolved oxygen; (4) particulate inorganic matter (PIM); (5) pH; (6) Chlorophyll-a; and (7) water flow velocity. Seven field surveys were conducted at 80 locations to collect geo-spatial environmental data, which were then used to determine HSI scores using habitat suitability functions. The model results showed that the areas suitable (HSI >0.50) for oyster settlement and growth were characterized by relatively high salinities, Chlorophyll-a, dissolved oxygen and pH values. In contrast, freshwater dominated estuaries and nearby coastal areas with high suspended sediment were found less suitable (HSI <0.50) for oysters. HSI model results were validated with observed oyster distribution data. There was strong correlation between the HSI calculated by the model and observed oyster densities (r = 0.87; n = 53), shell height (r = 0.95; n = 53) and their condition index (r = 0.98; n = 53). The good correspondence with field data enhances the applicability of the HSI model as a quantitative tool for evaluating the quality of a site for oyster restoration and culture.

## Introduction

Reef-forming oysters are habitat-structuring species in coastal and estuarine areas providing essential ecosystem goods and services to human society [1, 2, 3]. Both their reef structures and suspension-feeding behaviour exert large ecosystem influences [4]. Conservation and restoration of reef-forming oyster is therefore important to maintain ecosystem health and provide multiple ecosystem services including: (1) shoreline stabilization [5, 6, 7, 8]; (2) water quality regulation [9, 10, 11]; (3) ecosystem succession [12]; and (4) fisheries production [13, 14, 15, 16]. This implies also a sustainable management of these aquatic resources. To restore or create healthy oyster reefs, it is necessary to know the habitat requirements of the target species.

The intertidal rock oyster, *Saccostrea cucullata* is the dominant oyster species living along the south-eastern coast of Bangladesh, but the natural population is under great threat for habitat deterioration caused by recent developmental activities (e.g., Matarbari power plant project, LNG import terminal in Moheshkhali Island). At the same time, oyster reef development is considered to enhance coastal resilience in Bangladesh [17]. Successful and sustainable oyster reef development largely depends on the selection of suitable sites that support long-term growth and survival of oysters [18,19,20]. In fact, site selection for such approach is very challenging for the coastal zone of Bangladesh. The area is very dynamic and influenced by the annual monsoonal climate. To enhance survival and growth, one requires an understanding of the complex interactions between oysters and their environment [21]. Based on these complex relationships, a model was developed to determine suitable locations for oyster reef creation.

In the present paper, we developed a habitat suitability index (HSI) model for *S*. *cucullata* in Bangladesh that can be a useful tool for coastal resource managers. A HSI model provides spatially explicit information on the relative potential of a given area to support a particular species of interest [22, 23, 24]. Over 150 HSI models for wildlife species were published prior to 1990 [25], with many others developed since then. For oysters, the HSI efforts focus on their: (1) aquaculture [26, 27]; (2) fishery production [28, 29]; and (3) restoration [20, 30, 31, 32, 33, 34]. To determine the reliability and utility of an HSI model, a four-step process is used consisting of development, calibration, verification, and validation [34, 35, 36, 37, 38].

A comprehensive field monitoring program was initiated to quantify the forcing functions of the model covering all seasons along the entire south-east coast of Bangladesh. Then, based on experimental physiological data along with the data from literature, environmental factors were calibrated in order to develop the habitat suitability functions for each environmental parameter considered. Finally, model results were verified using an independent and spatially explicit population dataset. The aim of this study was to develop and test a spatially explicit HSI model for *Saccostrea cucullata* as a function of selected site characteristics that can be used to identify areas for oyster restoration and reef development.

## Materials and methods

### Study area

**Fig 1:**
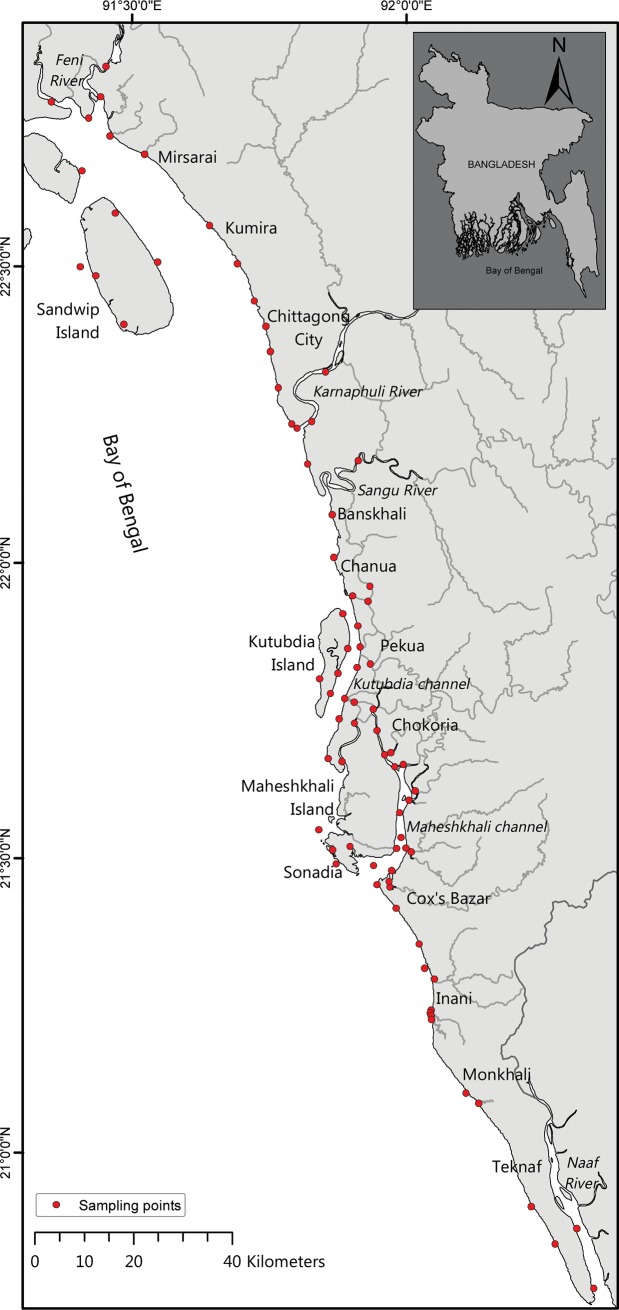
Geographical map showing the 80 field sampling locations (red dots) in this study for oyster habitat suitability model development in the South-eastern coastal waters of Bangladesh.

The present study was located in the south-eastern coastal waters of Bangladesh covering about 1,050 km of coastline including tidal river banks, from the Big Feni River in the west to the mouth of the Naaf River in the east (See Fig 1). The area consists of rivers, streams/tributaries, estuaries, channels, coastal waters and nearshore islands. No specific permissions were required for these locations/activities, as the field studies did not involve endangered or protected species. The northern part of the study area is a regular, unbroken stretch of coastline having intertidal mudflats and submerged sand banks. More to the south, a continuous sandy beach runs from Cox’s Bazar to the southern tip of the Teknaf peninsula. The coastal areas are characterised by a subtropical maritime climate. There are four seasonal weather patterns: (1) the dry winter season (December-February); (2) pre-monsoon (March-May); (3) monsoon (June-September); and (4) post-monsoon (October-November), which are principally governed by the southwest and northeast monsoon winds [39,40]. Among these four seasons, monsoon months are distinct from the non-monsoon months (see Table 1 as an example). About 80–90% of the annual rainfall is confined to the monsoon months, which makes the coastal environment very dynamic, with a lot of fluctuations in biotic and abiotic conditions [41,42,43]. During the season winter, the climate is mild and dry, with minimum air temperatures from 7–13°C and maximum temperatures from 24–31°C. The winds are predominantly north-easterly at the beginning of the winter and north-westerly at the end. May is generally the hottest month with air temperatures potentially reaching 40°C [44]. The heavy southwest monsoon rains begin in early June and continue into mid-October. During the monsoon period, floodwaters from extended rainfall pushes the freshwater to near the coast, while salinity variations in other seasons are relatively small [39]. The annual average rainfall varies from 1,500–3,500 mm [44]. Semi-diurnal tides are typical in these coastal waters, with a tidal range of approximately 3–6 m during the spring tide season [45]. Coastal water temperatures have distinct bimodal seasonal cycles with two warm and two cool seasons per year. Daily average water temperature is lowest (26.9°C) during winter months (December-January) and highest (29.7°C) during early summer months (April-May) [46]. Though the air temperature drops in winter for a short period, it has minor effect on seawater temperatures as it is buffered by the Bay of Bengal with its strong water circulations.

**Table 1:** Example of distinct environmental variation (mean ± standard deviation based on four observations during the monsoon period and three observations during the non-monsoon period) in monsoon and non-monsoon months (Location: Kutubdia channel;Year 2016). DO = Dissolved oxygen; Chla = Chlorophyll a; PIM = Particulate inorganic matter.

Major parameters	Monsoon	Non-monsoon	Annual mean
Water Temperature (°C)	29.4±0.2	28.1±1.6	28.5±1.4
Water salinity (ppt)	16.2±4.5	28±3.5	24.1±6.9
DO (% saturation)	72.2±4.9	77.1±5.3	75.5±5.4
pH (-)	7.7±0.0	8.0±0.1	7.9±0.1
Chla (μg l^-1^)	2.6±0.2	3.7±0.7	3.3±0.8
PIM (mg l^-1^)	571±84	240±108	351±190
Water flow velocity (m sec^-1^)	0.6±0.1	0.3±0.1	0.4±0.1
Total rainfall (mm) *	2162	726	2888

*Rainfall during 2016

### Assimilation of data sets on environmental variables

The most common variables utilised in HSI models for oysters are: temperature, salinity, pH, dissolved oxygen, water flow velocity, particulate inorganic matter (PIM), and chlorophyll-a (as a proxy of food for oysters, reviewed in [34]). For the present study, we collected environmental data from 80 sampling stations representing tributaries, river mouths, estuaries, channels and nearshore waters in south-eastern Bangladesh, covering about 1000 km of coastline (Fig 1). To cover seasonal influences, a total of 7 surveys visiting all 80 locations were conducted during the 12 month investigation period (January 2016 to December 2016). During the non-monsoon period (October–May), sampling was carried out only in representative months (January, May and November) covering three of the four seasons (i.e. winter, pre-monsoon and post monsoon). During the monsoon period (June—September), monthly sampling was carried out to quantify the variability during monsoonal period. Therefore, to reduce the high environmental variation, two s mean datasets (monsoon and rest of the seasons, here-after called non-monsoon) were created for model application.

Each survey was conducted during the full moon phase to cover the maximum tidal range and data were collected during flood and ebb tide periods to consider diurnal variations due to tides. Hand-held SCT (salinity, conductivity, temperature) and dissolved oxygen sensors (YSI model 30 and 55 respectively; YSI Inc., USA) were used to record water temperature (°C), salinity (ppt), dissolved oxygen (mg l^-1^).Water pH was recorded using a hand held pH meter (Model HI98107, HANNA Instruments, Romania). Water flow velocity (m sec^-1^) was measured by deploying a flowmeter (SKU 2030R; General Ocean Inc., USA) in mid flood and ebb tidal periods for 10 minutes. Concentration of total particulate matter (TPM, mg^-l^) was determined from water samples as weight of residue remaining on a filter (GF/C Whatman glass microfiber with 1.2μm pore size) after drying at 60°C for 12h. After ignition of TPM filter at 450°C for 5 h, particulate inorganic matter (PIM) concentrations were determined from weight loss. The chlorophyll-a concentration (*μ*g l^-1^) in water samples were determined by fluorescence meter (FluoroSense, Turner Designs, USA), calibrated by taking data from chlorophyll extraction into acetone following the procedure of Strickland and Parsons (1972) [47].

### Model description

**Fig 2:**
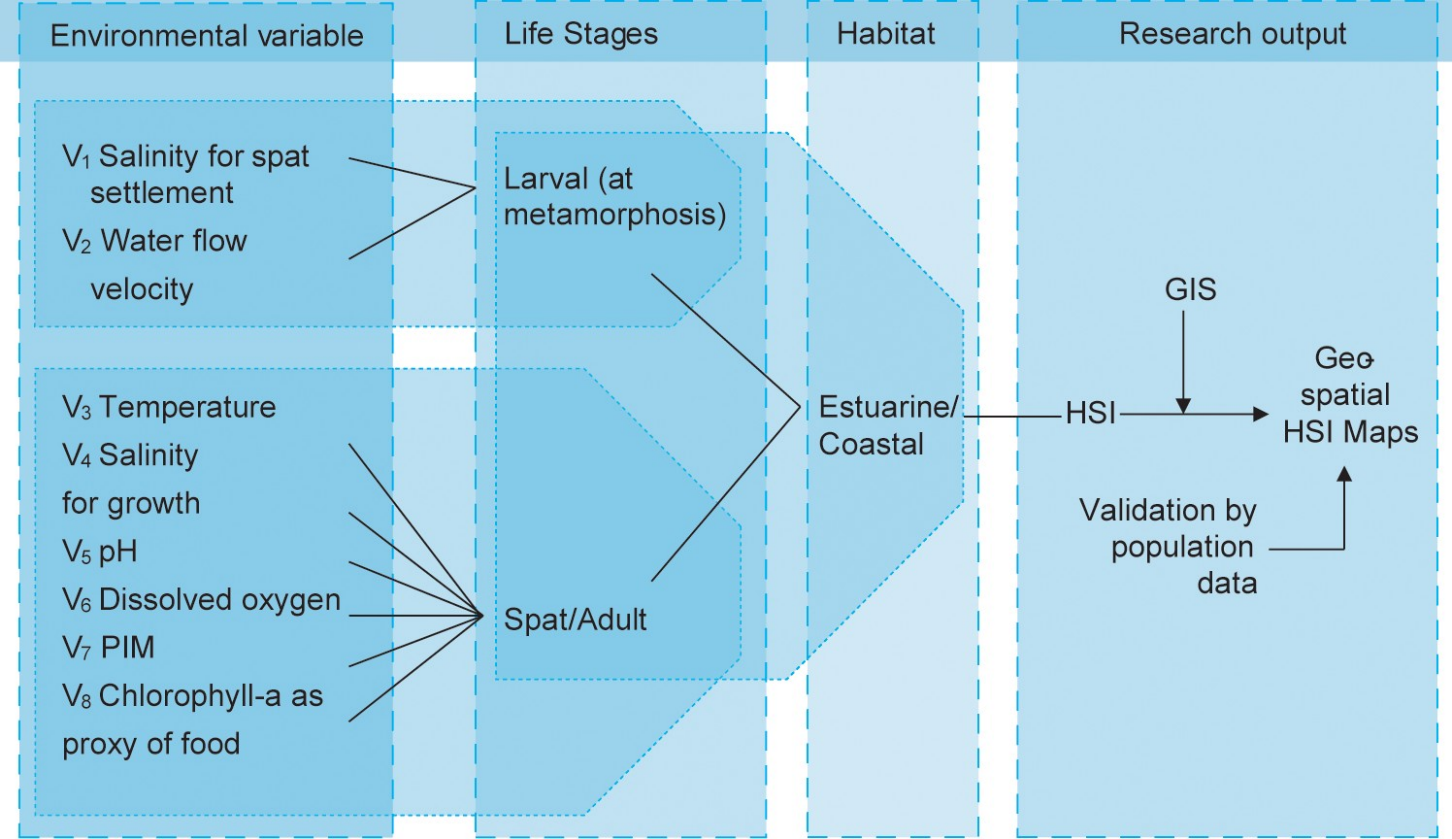
Tree diagram illustrating the relationships among environmental variables, life stages, and habitat types used to setup the Habitat Suitability Index (HSI) for the rock oyster *S*. *cucullata*.

The HSI model is composed of two life stage components: (1) the settling larval stage (at metamorphosis); and (2) the post-settlement life stages (spat and adult). Gametes, eggs and planktonic larval stages were excluded from the model as they have no habitat requirements beyond the water conditions which permits their parents to spawn. Fig 2 illustrates how the HSI is related to the variables and life stages of the oyster. The cycle starts at the metamorphosis, where the eyed-pediveliger larval stage that needs to settle onto a hard substrate. Ambient salinity, and the presence of suitable substrates are considered as key components for successful spatfall while high water flows can limit the settlement of oysters in turbulent waters. Temperature, salinity, pH, dissolved oxygen, PIM, and Chlorophyll-a were considered as important environmental variables for growth and survival of juveniles (= spat) and adult oysters.

**Fig 3:**
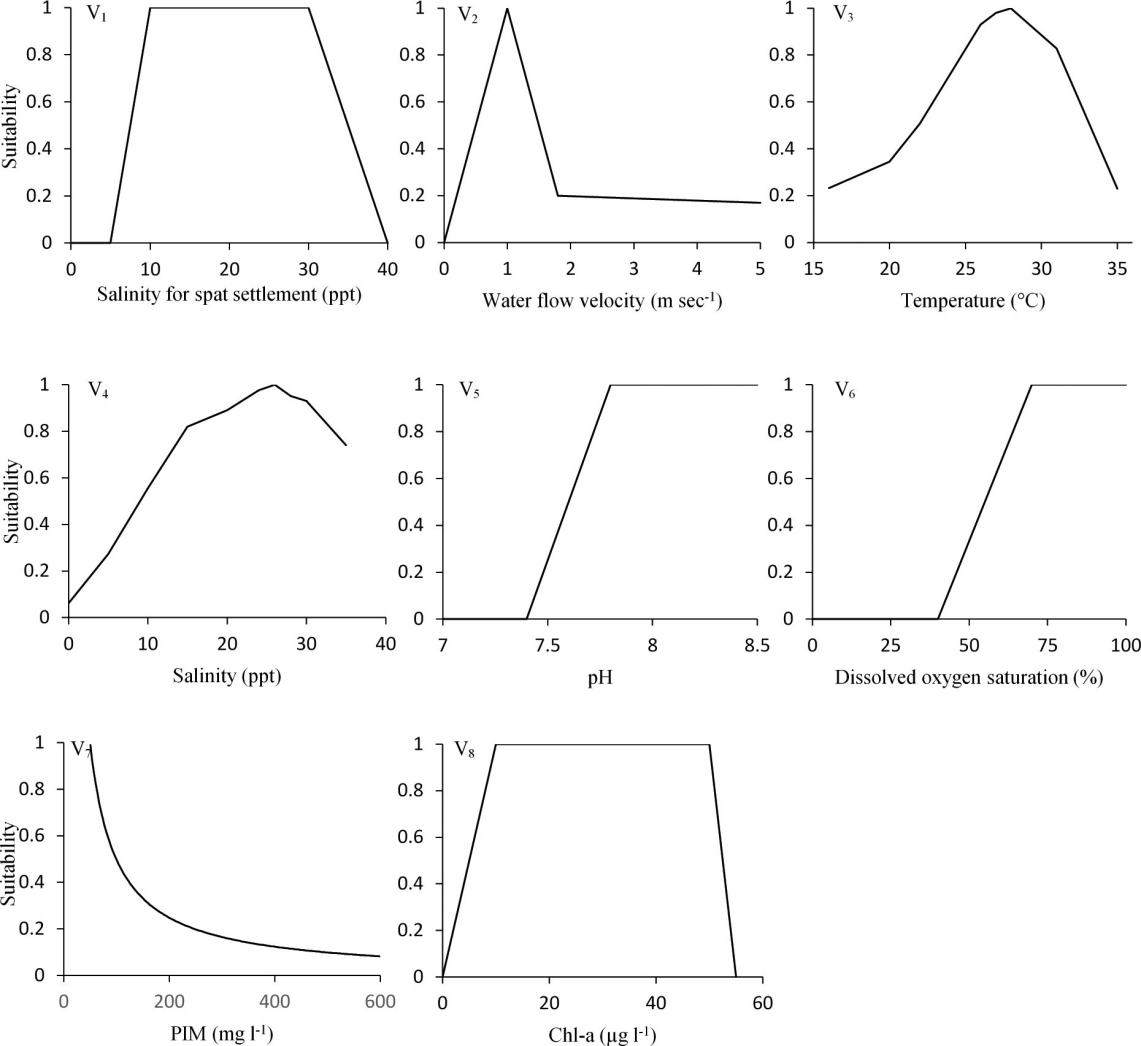
Relationships between environmental variables and associated habitat suitability values for the rock oyster *S*. *cucullata*. Top two graphs (V_1_, V_2_) were used for larval settlement, while the other graphs (V_3_-V_8_) were used for the post-settlement period in the model (see Table 2 for sources).

To calculate component indices for determining HSI, Suitability Index (SI) graphs were used that were obtained from existing literature (see Fig 3 and Table 2) except for salinity. Suitability Index (SI) graph for salinity are derived from empirical data from present study. Laboratory experiments were conducted to determine the influence of salinity (Fig 3D) on adult oyster respiration. Respiration rates were measured at 0, 5, 10, 15, 20, 25, 30, 35 ppt water salinities by keeping individual adult oysters (n = 12; Size = 5±0.2 cm) in closed chambers of 1 l capacity filled with water of 28 ± 0.5°C. Seawater was diluted by adding freshwater to get the desired salinity for the respiration experiments. Suitability scale was standardised from maximum respiration rates at observed salinity levels (i.e. maximum respiration rate = 1). Before running the respiration experiments, oysters were acclimatize for 24 h at the desired salinity condition to avoid stress related to change in physiological response. Respiration rates were measured when the oysters were actively filtering, which can easily be observed with shells open. Hand-held dissolved oxygen sensors (YSI model 55; YSI Inc., USA) were used to record the oxygen consumption rates at time intervals of five minutes, to check for a deviation in the linear decline. Each experimental trial was continued for about 2 hrs. Attention was given to prevent low oxygen concentration (< 3 mg O_2_ l^-1^) during trial.

**Table 2:** Data sources used to generate suitability index graphs for oysters.

Variables	Variables description	Data sources of suitability index (SI)	Conditions
Ss	Salinity for settlement	[28]	At eyed-pediveliger stage
V	Water flow velocity	[27]	-
T	Temperature	[21] Physiological relationship: Oxygen consumption rate vs temperature	Salinity: 28 ppt; pH: 7.9; Filtered seawater; Starved oysters
S_G_	Salinity for growth	This study Physiological relationship: Oxygen consumption rate vs salinity	Temperature: 28°C; pH: 7.9
pH	pH	[26]	-
DO	Dissolved oxygen	[26,27]	-
PIM	Particulate Inorganic Matter	[21] Physiological relationship: Water clearance rate vs suspended solids	Temperature: 28°C; Salinity: 28ppt; pH: 7.9
Chla	Chlorophyll-a as proxy for food	[26,27]	-

RR = -V*(pO_2,end_−pO_2,start_)/t, where RR = respiration rate in ml O_2_.h^-1^; V = volume of the chamber in l; pO_2,start_ and pO_2,end_ = oxygen concentration in ml l^-1^ at the start and at the end of the measurements; t = time difference in hour between start and end of the experiment.

SI is the Suitability Index for the environmental variables indicated in the Table 2. To obtain component index (CI) values for the two life stage components of the model, the SI values for appropriate variables were grouped and summarised by their geometric mean, as this is more sensitive to changes in individual variables than the arithmetic mean. It means that if an SI of 0 for any variable results in a CI of 0. Overall CI for settlement and post-settlement stages were estimated by using the following equations.

For the larval settlement:

$${CI}_{settlement-m}={\left({SI}_{Ss-m}\times {SI}_{V-m}\right)}^{1/2}$$

$${CI}_{settlement-nm}={\left({SI}_{Ss-nm}\times {SI}_{V-nm}\right)}^{1/2}$$

$${CI}_{settlement}=({CI}_{settlement-m}+{CI}_{settlement-nm})/2$$

For the post-settlement:

$${CI}_{post-settlement-m}={\left({SI}_{T-m}\times {SI}_{Sg-m}\times {SI}_{pH-m}\times {SI}_{DO-m}\times {SI}_{PIM-m}\times {SI}_{Chla-m}\right)}^{1/6}$$

$${CI}_{post-settlement-nm}={\left({SI}_{T-nm}\times {SI}_{Sg-nm}\times {SI}_{pH-nm}\times {SI}_{DO-nm}\times {SI}_{PIM-mn}\times {SI}_{Chla-nm}\right)}^{1/6}$$

$${CI}_{post-settlement}={\left({CI}_{post-settlement-m}\right)}^{1/3}{\times ({CI}_{post-settlement-nm})}^{1/3}$$

In these equations, CI _settlement_ is the component index for the larval settlement stage, which was considered for two seasonal component indices (i.e. CI _settlement-m_, CI _settlement-nm_) as the conditions for larval settlement can be different during the monsoon and non-monsoon periods. Thus, two different environmental mean data sets were used for the two periods (i.e. m = monsoon, nm = non-monsoon). During monsoon, a site may not be suitable for larval settlement, still it can have successful recruitment in the non-monsoon period. Therefore, arithmetic mean for seasonal larval CI is used instead of geometric mean, to consider the overall seasonal influences on the larval stage. Field observations indicated two seasonal settlement peaks in the investigated areas, thus equal weight coefficients were used for seasonal component indices (i.e., CI _settlement-m_ and CI _settlement-nm_). CI _post-settlement_ is the component index for the post-settlement (i.e. spat/adult) stage. Mean environmental data for monsoon and non-monsoonal were used as well to calculate CI_post-settlement_, as these seasons differ from each other. Based on the length of the seasonal periods (Monsoon = 4 months = 0.33 yr.; Non-monsoon = 8 months = 0.66 yr.), different weight coefficients were used for the seasonal component indices (i.e., CI _post-settlement-m_ and CI _post-settlement-nm_) in determining the component index for the post-settlement stage. In contrast to the component index for larval settlement, a multiplication function is used for the post-settlement phase because the habitat conditions need to be suitable throughout the entire year. After obtaining the mean environmental data, the suitability indices (SIs) were determined by using suitability graphs (Fig 3) and the component indices (CIs) were then calculated using the appropriate life stage equations. From the component indices, the overall HSI was determined following below equations as suggested by Cake [28]:

1. If the component index for the post-settlement stage (CI _post-settlement_) is the lowest component index (i.e., if CI _post-settlement_ < CI _settlement_), then *HSI* = *CI*_*post*−*settlement*_
2. If the component index for the post-settlement stage (CI _post settlement_) is not the lowest component index (i.e., CI _post-settlement_ > CI _settlement_), then *HSI* = (*CI*_*post*−*settlement*_×*CI*_*settlement*_)^1/2^

### Habitat suitability map

Habitat suitability indices were calculated for the 80 sampling locations along the south-east coast of Bangladesh using the measured environmental variables. To get a first estimate of the length of coastline that is suitable for oyster restoration, the HSI values of the 80 sampling locations were interpolated over the entire south-east coastline using a nearest neighbour algorithm [48]. For each HSI class, the total length (km) of the coastline was calculated using ArcGIS (version 10.5).

### Oyster data for model verification

To verify the model results with field observations, an oyster population survey was conducted after the monsoon. Based on the availability of substrates (jetty pillars, sluice gates, bridge pillars, and boulders), 53 sites among the 80 sampling locations were available for this survey. At the remaining sampling locations no nearby suitable substrates were present and therefore those sites were omitted from the analysis. Population data for model verification can be affected due to long sampling period during survey time, particularly for large scale area of the study. To avoid it, three voluntary teams simultaneously engaged at northern, middle and southern part of the study area and complete the filed survey within a week, covering only 2–3 stations in a day using speed boat. Oyster density, shell height and condition index (percentage of dry shell weight-dry flesh weight ratio) were determined by taking oyster samples at each site. For this, replicated (>5) quadrats (25 cm×25 cm) were used for sampling oysters from substrates available in the intertidal areas, which were positioned randomly along a 15 m long transect line (parallel to coastline) above mean lowest low water level (MLLW, ~ 0.5m), having similar emersion times for all locations. Quadrat areas without any oysters counted as zero. Quadrat areas with oysters were excavated without damaging the oysters. Living oysters were separated from dead shell remains. Living specimens were cleaned from epibionts and transported to the field laboratory, where individual shell height and fresh weight were measured. The soft tissue of each living oyster was separated from their shells, drained on paper towel and weighted after drying at 60°C for 12h. Geospatial oyster density data for the 53 locations were plotted on the potential HSI map where the size (area) of the circle represents the observed oyster density.

### Data analysis

Statistical differences in mean environmental variables for the monsoon vs the non-monsoon seasons were verified, using a simple t-test. Moreover, multiple linear regression models were used in order to relate the response variables (i.e. oyster density, shell height, and condition index,) to a set of independent variables (i.e. temperature, salinity, pH, dissolved oxygen, PIM, Chlorophyll-a, water velocity) recorded for the non-monsoon season. The non-monsoon season had more influence on settlement and growth, as oyster growth is almost stagnant during monsoon [21]. A forward stepwise procedure was followed by linear modelling to determine the environmental variable(s) that most influence oyster density, condition index, and shell height during growth season (i.e. non-monsoon months). Obtained data ranges for independent variables were checked whether they showed linear relationship with the suitability function curves used for the HSI modeling. Variance inflation factors (VIF) were used to check how much amount multicollinearity (correlation between independent variables) existed in a given regression analysis. The models were:

$$y_{d}=\beta_{0}+\beta_{1}x_{T}+\beta_{2}x_{S}+\beta_{3}x_{pH}+\beta_{4}x_{DO}+\beta_{5}x_{PIM}+\beta_{6}x_{Chla}+\beta_{7}x_{V}+\varepsilon_{d}$$

$$y_{h}=\beta_{0}+\beta_{1}x_{T}+\beta_{2}x_{S}+\beta_{3}x_{pH}+\beta_{4}x_{DO}+\beta_{5}x_{PIM}+\beta_{6}x_{Chla}+\beta_{7}x_{V}+\varepsilon_{h}$$

$$y_{CIndex}=\beta_{0}+\beta_{1}x_{T}+\beta_{2}x_{S}+\beta_{3}x_{pH}+\beta_{4}x_{DO}+\beta_{5}x_{PIM}+\beta_{6}x_{Chla}+\beta_{7}x_{V}+\varepsilon_{CIndex}$$

Where, *y* is the response variable indicating oyster density (d), shell height (h) and condition index (CIndex). *T* = temperature; *S* = salinity; *pH* = water pH; *DO* = dissolved oxygen; *PIM* = PIM; *Chla* = chlorophyll-a; *V* = water velocity. The parameter *β*_0_ is the *y*-intercept, which represents the theoretical expected value of *y* when each *x* is zero. The other parameters (*β*_1_, *β*_2_, …, *β*_7_) in the multiple regression equation are partial slopes. *β*_*j*_ (here, j = 1. . . .7) representing the expected change in *y* for a given unit increase in *x*_*j*_ while holding all other *x*s constant, and does not depend on the value of any other *x*. Other assumptions were: *E*(*ε*_*i*_) = 0 for all *i*, where *ε* is the residual terms of each model and i = 1….7 assigned for seven environmental parameters (i.e., T, S, pH, DO, PIM, Chla, and V) respectively; Var $(\varepsilon_{i})=\delta_{\varepsilon }^{2}$ for all *i*; the *ε*_*i*_*s* were independent; and *ε*_*i*_ was normally distributed. Before statistical analysis, the normality of a response and independent variables were tested using the Kolmogorov-Smirnov test and homogeneity of variances using Levene’s test. All analyses were performed using IBM SPSS statistics software (Version 2015) using α = 0.05.

## Results

### Environmental variables

**Fig 4:**
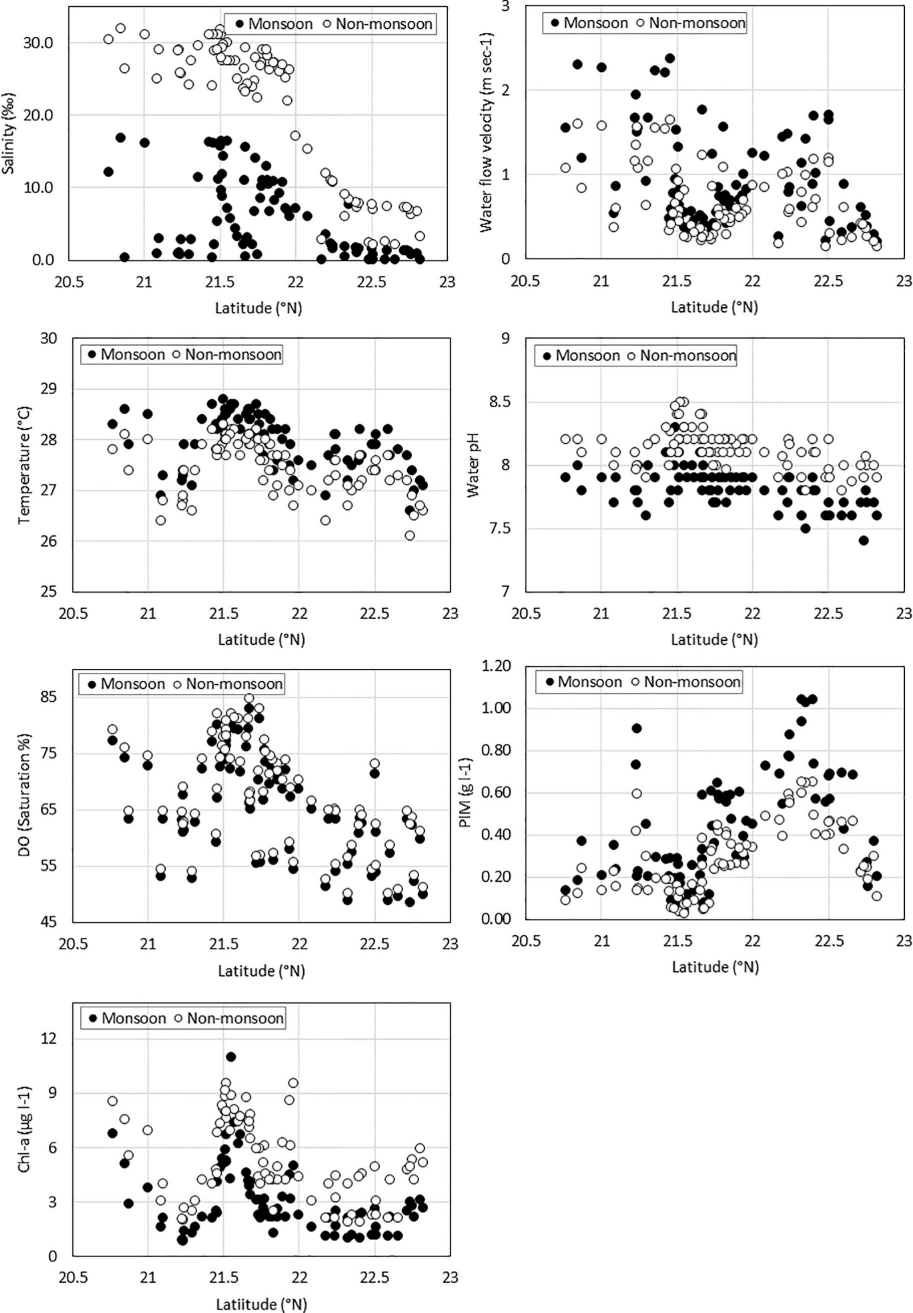
Environmental variables (salinity, water flow velocity, temperature, pH, dissolved oxygen, PIM, and chlorophyll-a) showed both spatial and seasonal (monsoon *vs* no-monsoon period) variations along the investigated sites. Sites are ordered from south to north (see Fig 1).

Environmental conditions showed both spatial and seasonal variations over the study period (Fig 4). A strong seasonal effect was observed, with monsoon months (June–September) differing from non-monsoon period (October–May). Particularly, salinity, PIM, chlorophyll-a concentrations and water flow velocity during monsoon period showed significant (p < 0.05) differences with the non-monsoon period. Spatially, a clear salinity gradient was observed for both seasonal periods showing an increasing trend from north to south. Feni, Mirsarai, and upper Chittagong coastal areas received strong influences from the nearby river systems and mean salinities ranged from 0.5–7.0 ppt with high mean particulate inorganic matter concentration (360–707 mg l^-1^). Mean salinities and suspended concentration in the lower part of Chittagong coast, Kutubdia, Moheshkhali, Cox’s Bazar, and Teknaf ranged from 6.7–29.5 ppt and 66–433 mg l^-1^, respectively. Among these areas, a few sites like Sonadia (southern Maheshkhali) and the Teknaf peninsula were strongly dominated by the Bay of Bengal, showing smaller variation even in monsoon months. Chlorophyll-a concentrations varied from 0.8–9.6 μgl^-1^ and was relatively high in the southern part of the study area compared to the freshwater dominated and turbid northern part of the study area. Mean water temperatures for all stations showed minimal variation (27–28.6°C) throughout the entire study period. Water pH levels ranged from 7.4–8.5 along the station sampled. pH was relatively high in non-monsoonal months and showed reduced values from north to south, probably influence of river discharge. Saturation level of dissolved oxygen varied from 49–91%. No significant differences (p = 0.70) in monsoon and non-monsoon season were observed for dissolved oxygen concentration, but a decreasing trend was observed from South to North which might be related to the organic loading from the rivers. Water flow velocity became stronger in monsoon periods and higher in exposed vs sheltered sites, ranging from 0.2–2.4 m sec^-1^.

### Model estimation

**Fig 5:**
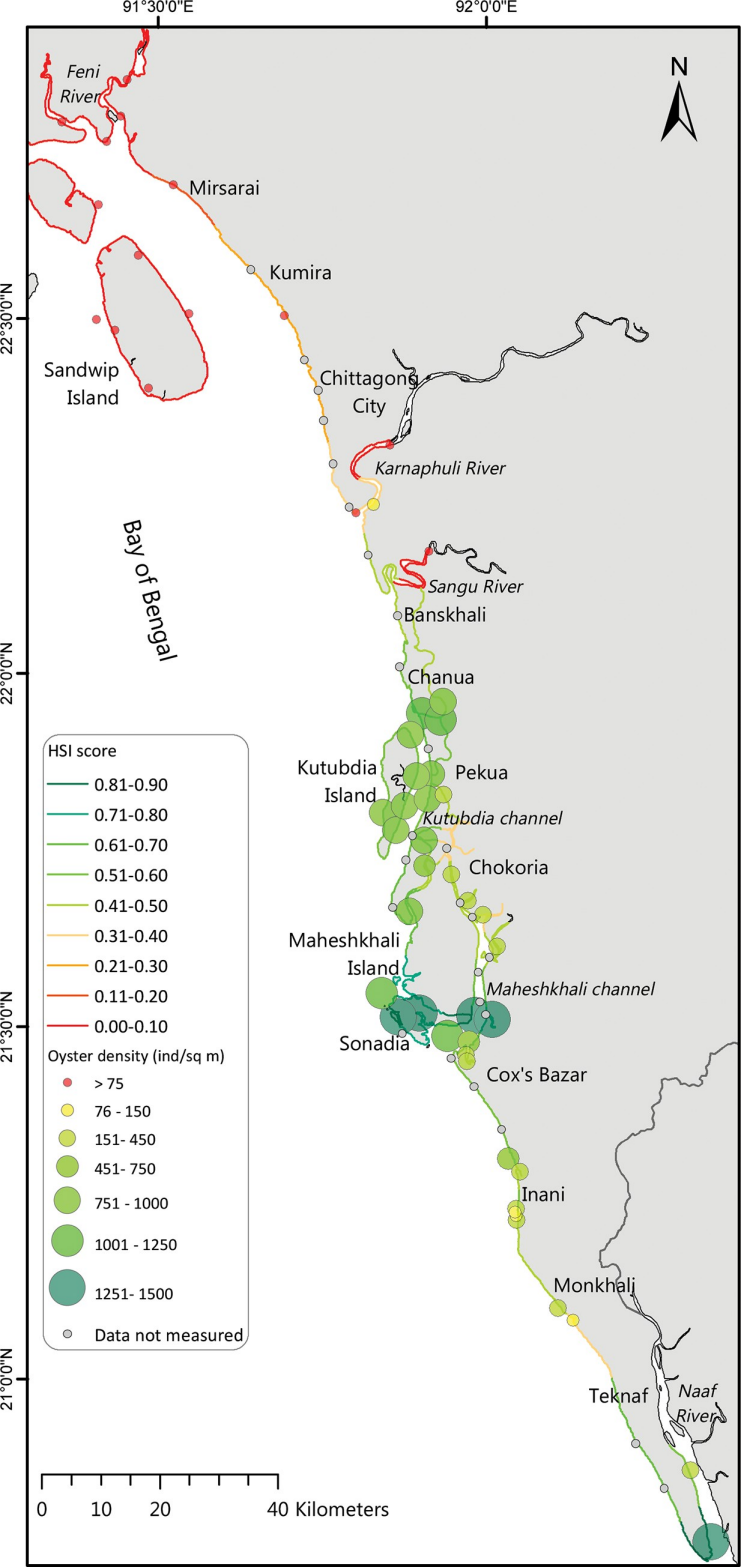
Map summarizing the results of the HSI indicating the suitability of the investigated sites (coloured lines), and verification of the model results with observed oyster density (coloured circles). Data not measured are sites (n = 27) where no substrate was available and therefore omitted from the oyster population survey.

By considering the effect of the two seasons, 37 sites, occupying approximately 397 km of coastline were predicted as suitable (HSI score >0.50) for year round growth of oysters (Table 3). Most of these sites were located in the area of lower Chittagong (Banskhali, Chanua), Pekua, Kutubdia, Moheshkhali, Sonadia, Cox’s Bazar and Teknaf coastal waters (Fig 5). Among those sites, 114 km of coastline along Sonadia, south-western Maheshkhali channel and southern tip of Teknaf peninsula were predicted as places with the highest HSI score (HSI score >0.7). In addition, 24 scattered sites in the southern part, representing a coastline of approximately 269 km, were less suitable for oysters (HSI score: 0.3–0.5). 19 sites representing approximately 391 km of coastline showed least prospect (HSI score: 0.0–0.3) for oyster development. Most of these sites belong to the coastline between Sandwip-Feni to mouth Karnaphully River. Few sites in the inner parts of the Moheshkhali channel, Chokoria and Cox’s Bazar coast (Inani and Monkhali) were also not considered as potential sites for oyster development. A habitat suitability map is presented in Fig 5 based on HSI scores.

**Table 3:** Estimated length of the coast corresponding with HSI scores.

HSI score	# of sites	Cumulative length of coast (km)
0.00–0.10	13	332
0.11–0.20	1	12
0.21–0.30	5	46
0.31–0.40	7	101
0.41–0.50	17	168
0.51–0.60	19	186
0.61–0.70	9	96
0.71–0.80	6	74
0.81–0.90	3	40
0.90–1.00	0	0

### Field verification

**Fig 6:**
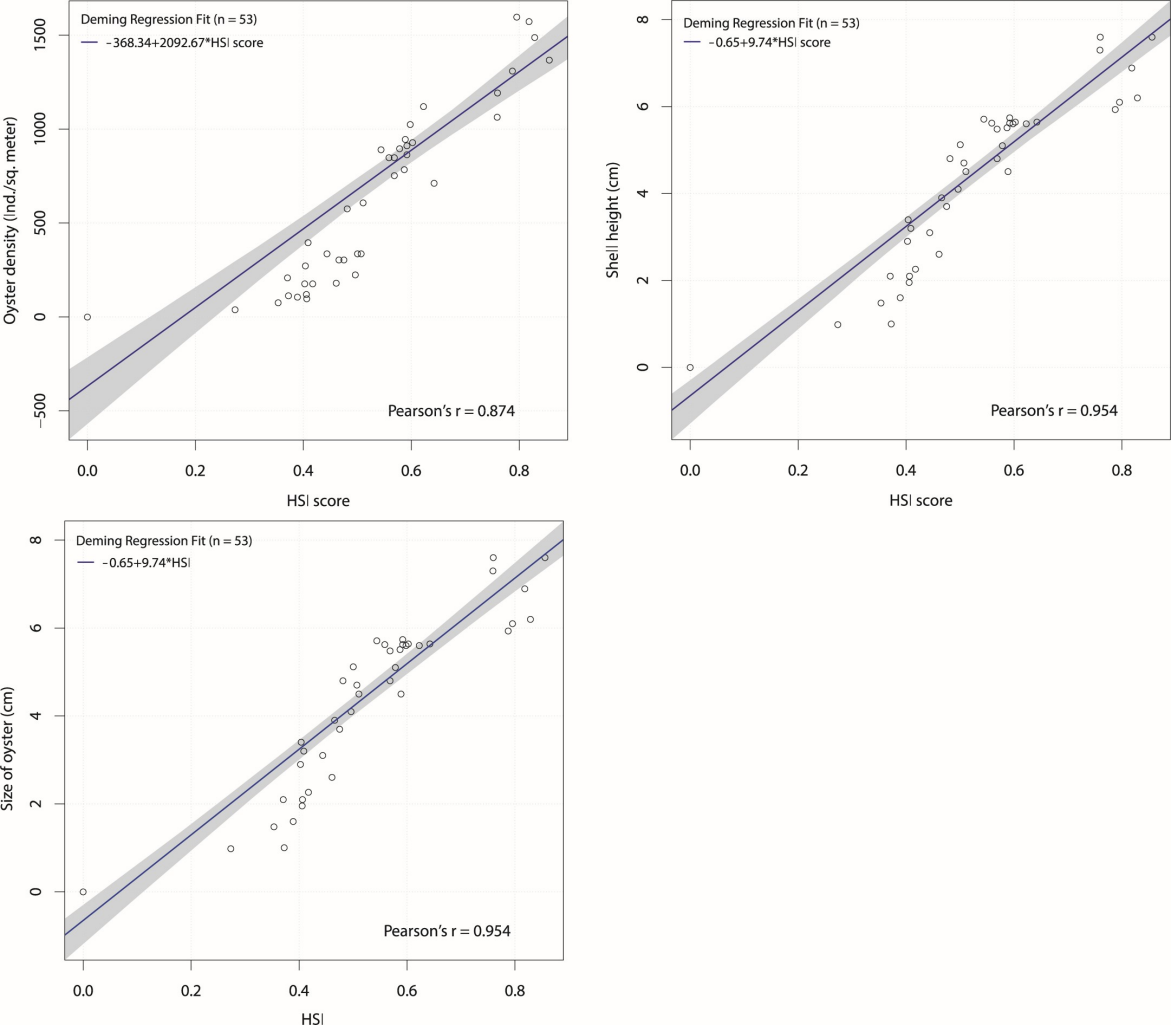
Habitat suitability index (HSI) scores derived from seven environmental datasets correlated against: live oyster density (top left), shell height (top right), condition index (bottom) shown with standard deviation (n = 53). The 0.95 confidence bounds (grey coloured) were calculated with the bootstrap (quantile) method.

**Fig 7:**
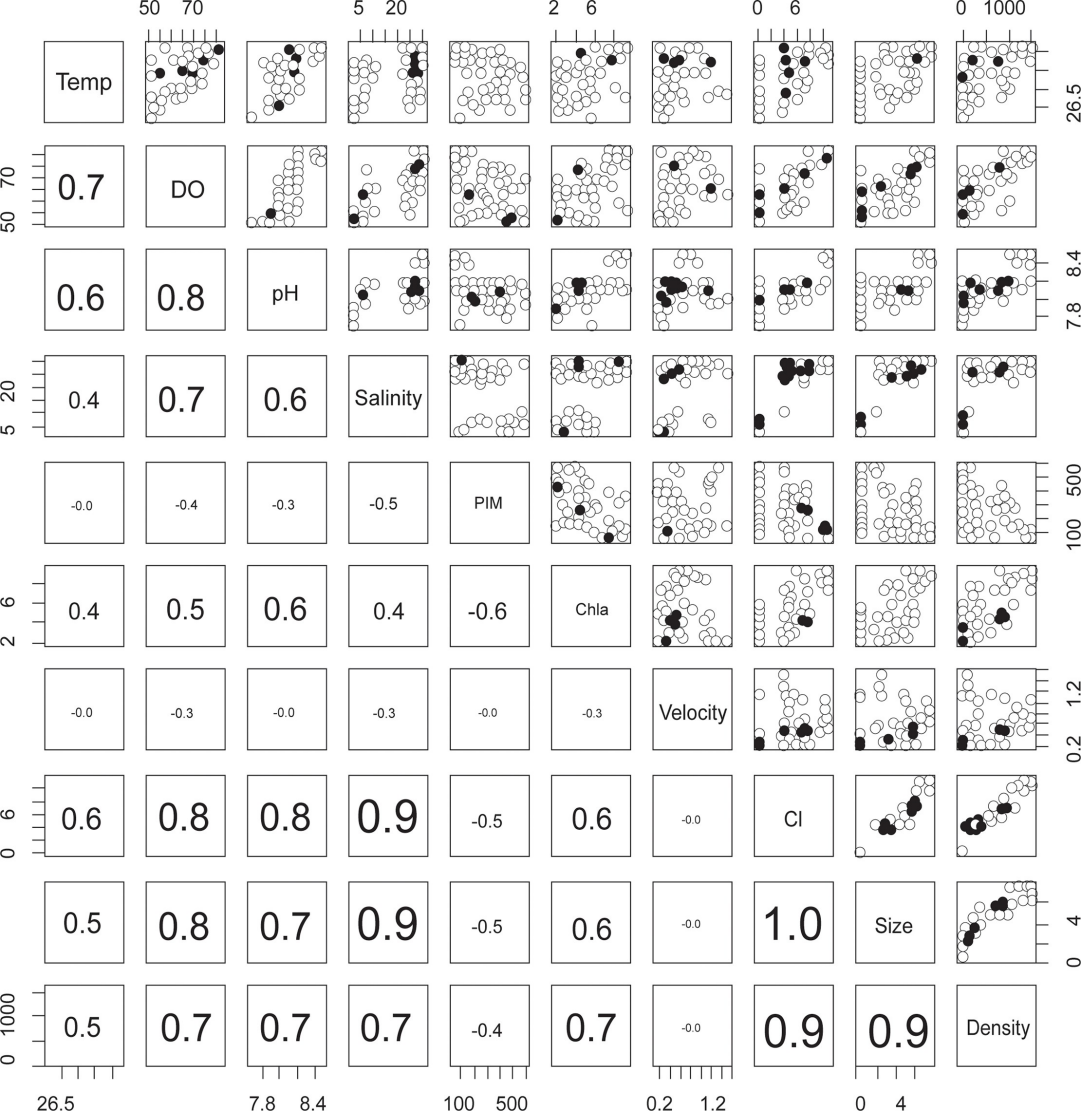
Scatter plots and correlation coefficients (correlations > 0.7 are shown in bold) among the independent (Temperature, dissolved oxygen, pH, salinity, PIM, chlorophyll-a, water flow rate) and dependant variables (CI, shell height, oyster density).

Three population descriptors were used for the assessment: (1) oyster density; (2) shell height; and (3) condition index. These were then correlated with the HSI scores for model verification (Fig 6). HSI values showed a strong positive relationship with the mean oyster densities (r = 0.87). Oysters were not observed at sites which had an HSI score less than 0.27. The highest number of oysters (1064–1596 indiv. m^-2^) were observed at sites which showed highest HSI scores (>0.70) (Fig 5). Moreover, mean oyster size (shell height) varied among the sites and showed positive relationship (r = 0.95) with HSI values as well. Variation in shell height was higher for upper HSI values, suggesting that the oysters in high HSI sites have multiple age classes due to multiple recruitments years. The oysters grew bigger in size (>5 cm shell height) in those sites, where the HSI score exceeded 0.50. Regression results for HSI and condition index also showed a similar trend. Field data showed that the shell-body flesh weight ratio largely varied (4.0–10.9%) among sites. Condition index increased with the increasing HSI scores, showing good correlation (r = 0.98). Condition indices were found relatively high (>6%), when the HSI score exceeded 0.50. Condition index for lower HSI sites showed more variability, which might be due to larger seasonal variation at these sites. Conversely, sites with high HIS values showed less variability in soft tissues coinciding with smaller seasonal variation. All the population descriptors were also strongly correlated (r > 0.90) with each other (Fig 7), thus showing good agreement with HSI scores.

### Influencing environmental factors

The linear regression model results indicated that salinity, chlorophyll-a, pH, and dissolved oxygen are the main predictors of oyster occurrence and their conditions (Table 4, for more details see S2 Table). Water temperature, PIM and water flow velocity were removed from the model during the stepwise procedure, as these factors failed to improve the model outputs. Salinity and Chlorophyll-a were found as common explanatory variables in the model that influenced oyster density, shell height and condition index. Oyster density and also shell height had high values in areas where the oxygen saturation level was relatively high. pH values also contributed to explain observed condition index of oysters. Scatter plots and correlation coefficients among all variables also gave the same results (Fig 7). Collinearity statistics in the linear model showed that variance inflation factors (VIF) were less than 5. It rejected the hypothesis of a multicollinearity relationship among environmental factors, thus explanatory variables used in the linear models were independent.

**Table 4:** Summary of linear regression models that were used to correlate among the dependant variables (oyster density, condition index and shell height) with independent environmental variables (for more details including the beta values of each predictors, see S2 Table).

Model	R	R Square	Adjusted R Square	Std. Error of the Estimate	Change Statistics			
					F	df1	df2	Sig. F
y_d_	0.839^a^	0.704	0.686	276.01538	8.216	1	49	0.006
y_CIndex_	0.926^b^	0.858	0.849	1.35382	6.250	1	49	0.016
y_h_	0.925^c^	0.855	0.846	0.97826	5.428	1	49	0.024

^a^ Predictors: Dissolved oxygen, Chl-a, Salinity; dependant variable: Oyster density

^b^ Predictors: Salinity, pH, Chl-a; dependant variable: Condition index

^c^ Predictors: Salinity, Chl-a, Dissolved oxygen; dependant variable: Mean shell height

## Discussion

Selection of relevant environmental variables for HSI model development is critical. It depends on the magnitude of the environmental factors related to habitat quality, as they vary in time (i.e. seasons) and space and the tolerance range of the oysters. In this study we developed an HSI model for the intertidal rock oyster, *S*. *cucullata* using seven environmental factors. Out of seven environmental factors, four factors viz., salinity, chlorophyll-a, dissolved oxygen and pH were found to be predictors of oyster density, condition index, and shell height. Water temperature, PIM concentrations and water flow velocity were not consider as predictors in the linear models used in this study. More than 70% of the variation in dependent descriptors (i.e. oyster density, condition index, and shell height) was explained by adding the variables: salinity, Chlorophyll-a, dissolved oxygen and pH (See S2 Table).

A large number of sites (n = 42) investigated in this study showed a decrease in salinity (> 5ppt) during the monsoon period. Most of these sites were located in upper south-eastern coast of Bangladesh, where large number of newly settled oysters die during the monsoon period. HSI scores were correspondingly low in these low salinity areas (S1 Table). Indeed, fluctuations in salinity regulate metabolic activities in oysters living in shallow marine and estuarine areas [49]. The reproductive capacities, spat settlement and growth of oysters are typically impaired by low salinities [50]. Low salinities can also cause mass mortalities of tropical oysters during the monsoon season, if the exposure to low salinities last too long [51]. In contrast, oysters flourished at sites of the investigated area where the salinity remained more than 10 ppt.

Chlorophyll-*a* concentrations varied both spatially and seasonally within the study area, and were lower during the monsoon period as compared to the non-monsoon period. Chlorophyll-*a* concentration increased with decreased suspended sediment load which might be due to better light penetration enhancing primary productivity. Higher HSI scores were found at locations where chlorophyll-a concentrations were high. In a study by Sasikumar et al. (2007) [52], oyster growth was positively correlation with Chlorophyll-*a* concentrations.

Dissolved oxygen levels appeared not to be critical in the investigated areas (all sites have values >50% saturation level). pH values were relatively high in the non-monsoon season as compared to the monsoon season. However, both parameters showed an increasing trend towards the south, which might be the influence of strong water circulation from the Bay of Bengal [46]. Oyster densities and related condition indices were relatively high in the area, where oxygen saturation levels (>70%) and pH (>7.9) also were high. In the Indian coasts, dissolved oxygen and water pH showed positive correlation with oyster spat settling rate [49,53]. Physiological activities slowed down with decreasing pH (<7.75) [54].

Water temperatures did not vary much along the coastline and did therefore not show any significant correlation with any dependent variables. PIM concentration (i.e. suspended sediment) in the study area varied between 21 and 1044 mg l^-1^ depending on the distance from river mouth. It showed a clear seasonal pattern at all sites with a large increase during the monsoon period, when 80 percent (~ 1850 mm) of the total rainfall occurs along with huge amounts of suspended sediments carried to the coast via rivers. Oysters can feed in turbid environments, but are less efficient and produce copious amount of pseudofaeces, which affects gill sorting [55, 56]. However, geo-spatial field PIM data did not show any significant correlation with the population descriptors used for model validation. Field observations also confirmed that *S*. *cucullata* population can thrive under high turbid (<700 mg l^-1^) conditions, if other environmental variables are optimal. Water flow velocity also did not show a good correlation with any of the dependent descriptors as the oysters were present in both high and low energy coasts, where other environmental factors are favourable. So, water temperature, PIM concentrations and water flow velocity can be neglected for determining the oyster habitat suitability in Bangladesh coast. In this regard, a sensitivity test was performed through simple model application, where these three factors were not included. It provided similar results (R^2^ = 0.96) in categorizing the site characteristics in terms of HSI scores.

Means of annual survey data are often used as input variables into HSI models [20, 27]. It may not provide appropriate HSI scores to evaluate a site, if strong seasonal influences exist as in monsoon dominated areas [24]. Some habitat factors can be constant over time (like in our case water temperature), while other factors viz., salinity, pH, PIM, dissolved oxygen, Chlorophyll-a may show strong seasonal differences. The use of extreme values for dynamic environmental variables in a HSI model can predict presence or absence of target species. However this approach might underestimate habitat quality, if the extreme values are not lethal to target species. Annual means without a seasonal considerations and also extreme environmental variable values were applied to evaluate the consequences in our model outputs. These provided high and low number of suitable sites respectively, which did not reflect field situations. Extreme values (i.e. observed lower ranges) only need to use, when they reach at or near the lethal levels and limit the survival. Otherwise mean seasonal data should consider to determine the component index of each environmental factor. Moreover, tolerance ranges could vary with different life history stages. Though adult oysters can tolerate extreme low salinities for extended periods, small periods of low salinity have a pronounced effect on settlement rate [57]. Spat settlement was generally unsuccessful during the monsoon period at many sites, but this phenomenon may not determine the quality of a habitat over the entire year. Spatfall after the monsoon is also important to maintain the population in dynamic coast. Particularly, the oysters that survive the non-monsoon period can then also survive the next monsoon months as they grow and tolerate low salinities. These seasonal and longer life stage considerations improved the model outputs with respect to previous unreported version of the model, and HSI scores showed strong correlations (r >0.87) with the oyster population descriptors (i.e. density, shell height and condition index). This study also assumed that all model inputs and functions were independent. However, one environmental factor can be influenced by others. For example, water pH can be regulated by salinity conditions as both the factors are influenced by the Bay of Bengal. This type of relationship should be considered to further improve the model.

Field verification is a critical part to determine the accuracy of HSI models, but is often lacking in oyster HSI models [34]. Adult oyster density is commonly used to validate an HSI model; however, this may not explain the complete picture. Here we not only considered adult oyster density, but also size (shell height) variation and condition index from 53 sites. All these descriptors demonstrate strong positive relationships with the HSI scores. Mean oyster size (> 5 cm) and condition index were to be high at those sites, where the HSI scores exceeded 0.50. In Bangladesh coastal waters, it usually takes more than a year to reach 5cm size (shell height) [21]. It confirms that oysters survive longer than a single year and that oyster populations probably can become self-sustaining with multiple year (size) classes when HSI scores are greater than 0.50. Population data were collected from a single survey after the monsoon period; this may differ with other seasons, yet it confirmed that oyster survival occurred after the monsoon. Still, demographic information for various seasons may improve the validation. Incorporation of the HSI scores into a GIS interface provides a visual aids in the format of maps for coastal resource managers and policy makers. An attempt was made to develop an HSI geospatial map for oysters, where validation data that reflect the population survey were used. We investigated about 1050 km of coastline using 80 representative sampling sites, and the strong spatial patterning in the map of HSI scores shows regions with good habitat suitability within the study area (see Fig 5). This gives a spatially explicit visualization of potential oyster habitats along the south-east Bangladesh coast. A simple nearest neighbour algorithm was used as an interpolation technique in a GIS interface to categorize the length of coastline using HSI scores. This forms a good basis for site selections, thus can be further expanded upon by increasing the number of sampling sites and the extent of the temporal environmental samples.

The present study has attempted to include all the available information to identify suitable sites for oysters through HSI model development. Nevertheless, the approach could be refined further with additional information. Such as, the amount of substrate available is important as it would also both contribute to the HSI and also could affect oyster density. There are some areas that showed potential for oyster development, but oysters were absent due to lack of substrate. Artificial hard substrate can be added there to test the model results. In this regard, bottom characteristics and wave energy conditions of coastal sites could be useful for determining the substrate types, which is inevitable for oyster reef formation. Yet, the verification of the model with field surveys shows a good fit for this oyster HSI model.

## Conclusion

This study developed an HSI model for the intertidal rock oyster, *Saccostrea cucullata* and applied it to the entire south-eastern coast of Bangladesh. Salinity, Chlorophyll-a, dissolved oxygen and pH were identified as driving factors that determine the habitat quality of oyster populations along this region. The results clearly show that freshwater dominated low saline estuaries and nearby coastal areas with high suspended sediments are least suitable for oyster settlement and growth. In contrast, the bay dominated areas with relative high salinity, Chlorophyll-a, dissolved oxygen and pH were found to be suitable for oyster settlement and growth. Seasons (i.e. monsoon and non-monsoon) and life stage (i.e. settlement and post settlement) considerations are found effective and suggested as integral part in habitat suitability model formulation for subtropical dynamic coastal systems. In this study, the HSI model results match the current distribution of oysters throughout the investigated area. The good correspondence with the field data enhances the reliability of the presented HSI model as a quantitative tool for planning oyster restoration and managing oyster resources along the south-eastern coast of Bangladesh.

## Supporting information

S1 Table Measured environmental conditions and oyster population characteristics for each HSI score (divided into 9 HSI classes), based 80 sampling stations (see Fig 1). (DOCX)

S2 Table Summary of linear model results. (DOCX)

## References

[1] Beck MB, Brumbaugh RD, Airoldi L, Caranza A, Coen LD, Crawford C, et al Shellfish Reefs at Risk: A Global Analysis of Problems and Solutions. Virginia: The Nature Conservancy; 2009.

[2] Beck MW, Brumbaugh RD, Airoldi L, Carranza A, Coen LD, Crawford C, et al Oyster reefs at risk and recommendations for conservation, restoration, and management. Bioscience 2011;61: 107–116.

[3] Coen LD, Brumbaugh RD, Bushek D, Grizzle R, Luckenbach MW, Posey MH, et al Ecosystem services related to oyster restoration. Mar Ecol Prog Ser. 2007;341: 303–307.

[4] Newell RIE. Ecosystem influences of natural and cultivated populations of suspension-feeding bivalve molluscs: A review. J Shellfish Res. 2004;23, 51–61.

[5] Piazza BP, Banks PD, La Peyre MK. The potential for created oyster shell reefs as a sustainable shoreline protection strategy in Louisiana. Restoration Ecol. 2005;13: 499–506.

[6] Scyphers SB, Powers SP, Heck KH, Byron D. Oyster reefs as natural breakwaters mitigate shoreline loss and facilitate fisheries. PLoS ONE 2011; 6: e22396 10.1371/journal.pone.0022396 21850223 PMC3151262

[7] Walles B, De Paiva JS, Van Prooijen B, Ysebaert T, Smaal A. The ecosystem engineer *Crassostrea gigas* affects tidal flat morphology beyond the boundary of their reef structures. Estuaries Coasts 2015;38: 941–950

[8] Ysebaert T, Walles B, Dorsch C, Dijkstra J, Troost K, Volp N, et al Ecodynamic solutions for the protection of intertidal habitats: the use of oyster reefs. J Shellfish Res. 2012;31: 362–362.

[9] Kellogg ML, Cornwell JC, Owens MS, Paynter KT. Denitrification and nutrient assimilation on a restored oyster reef. Mar Ecol Prog Ser. 2013;480: 1–19.

[10] Newell RIE, Cornwell JC, Owens MS. Influence of simulated bivalve biodeposition and microphyto-benthos on sediment nitrogen dynamics: A laboratory study. Limnol Oceanogr. 2002;47: 1367–1379.

[11] Piehler MF, Smyth AR. Habitat-specific distinctions in estuarine denitrification affect both ecosystem function and services. Ecosphere 2011; 2:article 12 10.1890/ES10-00082.1

[12] La Peyre MK, Miller LS, Miller S, Melancon E. Comparison of Oyster Populations, Shoreline Protection Service, and Site Characteristics at Seven Created Fringing Reefs in Louisiana: Key Parameters and Responses to Consider In: Bilkovic DM, Mttchell MM, La Peyre MK, Toft JD, editors. Living Shorelines: The Science and Management of Nature-Based Coastal Protection. CRC Press (Taylor & Francis Group); 2017 pp. 363–381.

[13] Peterson CH, Grabowski JH, Powers SP. Estimated enhancement of fish production resulting from restoring oyster reef habitat: quantitative valuation. Mar Ecol Prog Ser. 2003;264: 249–264.

[14] Gregalis KC, Johnson MW, Powers SP. Restored oyster reef location and design affect responses of resident and transient Fish, crab, and shellfish species in Mobile bay, Alabama. Transact Am Fish Soc. 2009;138: 314–327.

[15] Tolly SG, Volety AK. The role of oysters in habitat use of oyster reefs by resident fishes and decapods crustaceans. J Shellfish Dis. 2005;24: 1007–1012.

[16] Hossain MS, Rothuis A, Chowdhury SR, Smaal A, Ysebaert T, Sharifuzzaman SM, et al Oyster aquaculture for coastal defense with food production in Bangladesh. Aquacult Asia 2013;18(1): 15–24.

[17] Hossain MS, Chowdhury SR, Navera UK, Hossain MAR, Imam DI, Sharifuzzaman SM. Opportunities and Strategies for Ocean and River Resources Management Dhaka: Background paper for preparation of the 7th Five Year Plan. Planning Commission, Ministry of Planning, Bangladesh; 2014.

[18] Hargis WJJ, Haven DS. Chesapeake Bay oyster reefs, their importance, destruction and guidelines for restoring them In: Luckenbach MW, Mann R, Wesson JA, editors. Oyster Reef Habitat Restoration: A Synopsis and Synthesis of Approaches. Gloucester Point: Virginia Institute of Marine Science Press; 1999 pp. 329–358.

[19] Schulte DM, Burke RP, Lipcius RN. Unprecedented restoration of a native oyster metapopulation. Science 2009;325: 1124–1128. 10.1126/science.1176516 19644073

[20] Pollack J, Cleveland A, Palmer T, Reisinger A, Montagna P. A restoration suitability index model for the eastern oyster Crassostrea virginica in the Mission-Aransas Estuary, TX, USA. PLoS ONE 2012; 7, e40839 10.1371/journal.pone.0040839 22792410 PMC3394728

[21] Chowdhury MSN, Wijsman WM, Hossain MS, Ysebaert T, Smaal AC. 2018. DEB parameter estimation for *Saccostrea cucullata* (Born), an intertidal rock oyster in the Northern Bay of Bengal. J Sea Res. 2018; (under review). 10.1016/j.seares.2017.02.005

[22] US Fish and Wildlife Service. Standards for the Development of Habitat Suitability Index Models. Technical Report 103ESM, USDI Fish and Wildlife Service, Division of Ecological Services, Washington DC; 1981.

[23] Prosser DJ, Brooks RP. A verified Habitat Suitability Index for the Lousiana water thrush. J Field Ornithol. 1998;69: 288–298.

[24] Roloff GJ, Kernohan BJ. Evaluating reliability of habitat suitability index models. Wildl Soc Bull. 1999;27: 973–985.

[25] Terrell JW, Carpenter J. Selected habitat suitability index model evaluations. USGS/BRD/ITR-1997-0005. U.S. Geological Survey, Washington DC; 1997.

[26] Brown J, Hartwick E. A habitat suitability index model for suspended tray culture of the Pacific oyster, Crassostrea gigas Thunberg. Aquac Res. 1988;19: 109–126.

[27] Cho Y, Lee WC, Hong S, Kim HC, Kim JB. GIS based suitable site selection using habitat suitability index for oyster farms in Geoje-Hansan Bay, Korea. Ocean Coast Manage. 2012;56: 10–16.

[28] Cake E. Habitat suitability index models: Gulf of Mexico American Oyster. US Dept. Int. Fish Wildl. Serv. FWS/OBS -82/10.57; 1983.

[29] Soniat TM, Brody MS. Field validation of a habitat suitability index model for the American oyster. Estuaries 1988;11: 87–95.

[30] Barnes T, Volety A, Chartier K, Mazzotti F, Pearlstine L. A habitat suitability index model for the eastern oyster *Crassostrea virginica*, a tool for restoration of the Caloosahatchee Estuary, Florida. J Shellfish Res. 2007;26: 949–959.

[31] Soniat TM, Conzelmann CP, Byrd JD, Roszell DP, Bridevaux JL, Suir KJ, et al Predicting the effects of proposed Mississippi River diversions on oyster habitat quality: application of an oyster habitat suitability index model. J Shellfish Res. 2013;32: 629–638.

[32] Starke A, Levinton J, Doall M. Restoration of *Crassostrea virginica* (gmelin) to the Hudson River, USA: a spatiotemporal modeling approach. J Shellfish Res. 2011;30: 671–684.

[33] Swannack TM, Reif M, Soniat TM. A robust, spatially explicit model for identifying oyster restoration sites: case studies on the Atlantic and Gulf coasts. J Shellfish Res. 2014;33: 395 408.

[34] Theuerkauf SJ, Lipcius RN. Quantitative Validation of a Habitat Suitability Index for Oyster Restoration. Front Mar Sci. 2016;3: 1–9.

[35] Brooks RP. Improving habitat suitability index models. Wildl Soc Bull. 1997;25: 163–167.

[36] Tirpak JM, Jones-Farrand D, Thompson FR, Twedt DJ, Baxter CK, Fitzgerald JA, et al Assessing eco regional-scale habitat suitability index models for priority land birds. J Wildl Manage. 2009;73: 1307–1315.

[37] Cole CA, Smith RL. Habitat suitability indices for monitoring wildlife populations—an evaluation. Trans. North Am. Wildlife Nat Resour Conf. 1983;48: 367–375.

[38] Reiley BM, Bednarz JC, Brown JD. A test of the Swainson’s warbler habitat suitability index model. Wildl Soc Bull. 2014;38: 297–304.

[39] Mahmood N, Chowdhury NJU, Hossain MM, Haider SMB, Chowdhury SR. Bangladesh. In: Holmgren S, editor. An Environmental Assessment of Bay of Bengal Region. BOBP/REP/67; 1994 pp. 75–94.

[40] Khatun MA, Rashid MB, Hygen HO. Climate of Bangladesh. MET Report No. 08/2016. Dhaka: Norwegian Meteorological Institute and Bangladesh Meteorological Department; 2016.

[41] ESCAP, 1988. Coastal Environmental Management Plan for Bangladesh. Bangkok: Economic and Social Commission for Asia and the Pacific; 1888.

[42] Mahtab FU. Effect of climate change and sea-level rise on Bangladesh Report to Expert Group on Climate Change and Sea-level Rise. London: Commonwealth Secretariat; 1989.

[43] Pernetta JC. Marine Protected Area Needs in the South Asian Seas Region (Volume 1: Bangladesh). Gland: IUCN; 1993.

[44] BMD (Bangladesh Meteorological Department). Record book of meteorological data. Dhaka: Bangladesh Meteorological Department; 2017.

[45] BIWTA (Bangladesh Inland Water Transport Authority). Bangladesh Tide Tables. Dhaka: Department of Hydrology, BIWTA; 2017.

[46] Chowdhury SR, Hossain MS, Shamsuddoha M, Khan SMMH. Coastal Fishers’ Livelihood in Peril: Sea Surface Temperature and Tropical Cyclones in Bangladesh. Dhaka: Foreign and Commonwealth Office through British High Commission and Centre for Participatory Research and Development (CPRD); 2012.

[47] Stickland JDH, Parsons TR. A practical handbook of seawater analysis (2^nd^ edition). Ottawa: Fisheries Research Board of Canada; 1972.

[48] ESRI. Create Thiessen Polygons. 2017. Available from: http://pro.arcgis.com/en/pro-app/tool-reference/analysis/create-thiessen-polygons.htm#L_ (Accessed in November 23, 2017)

[49] Naik GM, Gowda G. Influence of environmental factors on oyster: A review. Int J Adv Sci and Tech Res. 2013;3(2): 341–353

[50] Rao KV. Observations on the probable effects of salinity on the spawning, development and setting of the Indian backwater oyster *Ostrea madrasensis* Preston. Proc Indian Acad Sci. 1951;33: 231–256.

[51] Angell CL. The biology and culture of tropical oysters. Philippines: ICLARM; 1986.

[52] Sasikumar G, Krishnakumar PK, Thomas S, Sampathkumar G, Nagaraja D, Bhat GS. Influence of environmental factors on growth rate of *Crassostrea madrasensis* (Preston) in suspended culture. Asian Fish Sci. 2007;20: 247–255

[53] Naik G. Influence of environmental factors on the oyster beds of Mulky estuary, south west coast of India. Ph.D Thesis, Karnataka Veterinary, Animal and Fisheries Sciences University, Bidar; 2012.

[54] Mahadevan S, Nayar KN. Ecology of oyster beds. CMFRI Bull. 1987;38: 7–13.

[55] Urban ER, Kirchman DL. Effect of kaolinite clay on the feeding-activity of the Eastern oyster *Crassostrea virginica* (Gremlin). J Exp Mar Biol Ecol. 1992;160: 47–60.

[56] Ward JE, Levinton JS, Shumway SE, Cucci T. Particle sorting in bivalves: in vivo determination of the pallial organs of selection. Mar Biol. 1998;131: 283–292.

[57] Hopkins AE. Attachment of larvae of the Olympia oyster, *Ostrea lurida*, to plane surfaces. Ecology 1935;16: 82–87.

