# Supplementary material for: A verified habitat suitability model for the intertidal rock oyster, Saccostrea cucullata
Source: PLoS One. 2019 Jun 11;14(6):e0217688. doi: 10.1371/journal.pone.0217688 (PMC6559651; doi:10.1371/journal.pone.0217688)
Supplement: S1 Table — (DOCX) [file pone.0217688.s001.docx]

**S1 Table. Measured environmental conditions and oyster population characteristics for each HSI score (divided into 9 HSI classes), based 80 sampling stations (see Fig 1).**

| HSI | # of site | Water Temp (°C) | | DO (Saturation %) | | pH  (-) | | Salinity  (ppt) | | PIM  (mg l-1) | | Chl-a  (µg/l) | | Water flow velocity (m/sec) | | Density (ind./m-2) | CI | Size (cm) | | |
| --- | --- | --- | --- | --- | --- | --- | --- | --- | --- | --- | --- | --- | --- | --- | --- | --- | --- | --- | --- | --- |
|  |  | M | NM | M | NM | M | NM | M | NM | M | NM | M | NM | M | NM |  |  | Min | Max |  |
| 0-0.10 | 13 | 27.5 | 27.0 | 55 | 56 | 7.67 | 7.96 | 0.5 | 4.1 | 506 | 370 | 1.82 | 3.48 | 0.59 | 0.41 | 0 | 0 |  |  |  |
| 0.11-0.20 | 1 | 26.6 | 26.1 | 58 | 63 | 7.7 | 7.66 | 0.1 | 5.3 | 239 | 250 | 2.5 | 4.85 | 0.57 | 0.41 | 0 | 0 |  |  |  |
| 0.21-0.30 | 5 | 27.6 | 27.1 | 61 | 63 | 7.76 | 8.06 | 1.5 | 7.1 | 748 | 498 | 2.14 | 4.08 | 1.17 | 0.81 | 0 | 0 |  |  |  |
| 0.31-0.40 | 7 | 27.8 | 27.3 | 58 | 60 | 7.75 | 8.05 | 1.4 | 15.3 | 680 | 459 | 1.98 | 3.74 | 0.79 | 0.55 | 125 | 4.0 | 0.82 | 3.25 |  |
| 0.41-0.50 | 17 | 27.9 | 27.4 | 64 | 66 | 7.83 | 8.11 | 2.9 | 23.8 | 362 | 233 | 2.82 | 4.83 | 0.83 | 0.61 | 263 | 4.5 | 0.79 | 4.93 |  |
| 0.51-0.60 | 19 | 28.0 | 27.5 | 69 | 71 | 7.87 | 8.15 | 8.9 | 26.5 | 435 | 271 | 2.83 | 5.09 | 1.13 | 0.79 | 772 | 6.5 | 0.8 | 8.59 |  |
| 0.61-0.70 | 9 | 28.3 | 27.8 | 74 | 76 | 7.95 | 8.22 | 11.3 | 27.2 | 241 | 181 | 3.97 | 6.85 | 1.21 | 0.84 | 920 | 7.3 | 0.77 | 9.01 |  |
| 0.71-0.80 | 6 | 28.5 | 28.0 | 76 | 78 | 8.06 | 8.34 | 13.2 | 29.4 | 211 | 128 | 6.06 | 8.67 | 0.94 | 0.72 | 1290 | 10.1 | 0.82 | 12.64 |  |
| 0.81-0.90 | 3 | 28.4 | 27.9 | 78 | 80 | 8.1 | 8.36 | 12.4 | 28.7 | 100 | 66 | 7.66 | 8.35 | 0.97 | 0.87 | 1476 | 10.9 | 0.86 | 12.82 |  |

Note: [Environmental variables are these averaged values over the number of sites per HSI class and over the representative months for monsoon and non-monsoon, while oyster population descriptors are averaged values over the number of sites per HSI class.].
