# Supplementary material for: A verified habitat suitability model for the intertidal rock oyster, Saccostrea cucullata
Source: PLoS One. 2019 Jun 11;14(6):e0217688. doi: 10.1371/journal.pone.0217688 (PMC6559651; doi:10.1371/journal.pone.0217688)
Supplement: S2 Table — (DOCX) [file pone.0217688.s002.docx]

**S2 Table. Summary of linear model results**

| Model, *y_d_* | | | | | | | | | | | | | | | | |  |
| --- | --- | --- | --- | --- | --- | --- | --- | --- | --- | --- | --- | --- | --- | --- | --- | --- | --- |
| Model | | R | R^2^ | | Adjusted R^2^ | | Std. Error of the Estimate | Change Statistics | | | | | | | | |  |
|  |  |  |  |  |  |  |  | R Square Change | | F Change | | df1 | | df2 | Sig. F Change | |  |
| 1 | | .749^a^ | .561 | | .552 | | 329.54381 | .561 | | 65.159 | | 1 | | 51 | .000 | |  |
| 2 | | .809^b^ | .654 | | .641 | | 295.26137 | .094 | | 13.531 | | 1 | | 50 | .001 | |  |
| 3 | | .839^c^ | .704 | | .686 | | 276.01538 | .050 | | 8.216 | | 1 | | 49 | .006 | |  |
| ^a^ Predictors: (Constant), DO | | | | | | | | | | | | | | | | |  |
| ^b^ Predictors: (Constant), DO, Chl-a | | | | | | | | | | | | | | | | |  |
| ^c^ Predictors: (Constant), DO, Chl-a, Salinity | | | | | | | | | | | | | | | | |  |
| ^d^ Dependent Variable: Oyster density | | | | | | | | | | | | | | | | |  |
| Coefficients^a^ | | | | | | | | | | | | | | | | |  |
| Model | | | | Unstandardized Coefficients | | | | | Standardized Coefficients | | t | | Sig. | | |  |  |
|  |  |  |  | B | | Std. Error | | | Beta | |  |  |  |  |  |  |  |
| 1 | (Constant) | | | -2070.583 | | 320.611 | | |  | | -6.458 | | .000 | | |  |  |
|  | DO | | | 39.133 | | 4.848 | | | .749 | | 8.072 | | .000 | | |  |  |
| 2 | (Constant) | | | -1779.843 | | 297.933 | | |  | | -5.974 | | .000 | | |  |  |
|  | DO | | | 28.771 | | 5.177 | | | .551 | | 5.557 | | .000 | | |  |  |
|  | Chla | | | 76.363 | | 20.760 | | | .364 | | 3.678 | | .001 | | |  |  |
| 3 | (Constant) | | | -1373.625 | | 312.497 | | |  | | -4.396 | | .000 | | |  |  |
|  | DO | | | 18.322 | | 6.059 | | | .351 | | 3.024 | | .004 | | |  |  |
|  | Chl-a | | | 71.397 | | 19.484 | | | .341 | | 3.664 | | .001 | | |  |  |
|  | Salinity | | | 14.745 | | 5.144 | | | .309 | | 2.866 | | .006 | | |  |  |
| ^a.^ Dependent Variable: Oyster density | | | | | | | | | | | | | | | | | |

**S2 Table. Summary of linear model results (continued)**

| Model, *y_CIndex_* | | | | | | | | | | | | | | | | |  |
| --- | --- | --- | --- | --- | --- | --- | --- | --- | --- | --- | --- | --- | --- | --- | --- | --- | --- |
| Model | | R | R^2^ | | Adjusted R^2^ | | Std. Error of the Estimate | Change Statistics | | | | | | | | |  |
|  |  |  |  |  |  |  |  | R Square Change | | F Change | | df1 | | df2 | Sig. F Change | |  |
| 1 | | .866^a^ | .750 | | .745 | | 1.76219 | .750 | | 152.793 | | 1 | | 51 | .000 | |  |
| 2 | | .917^b^ | .840 | | .834 | | 1.42313 | .090 | | 28.196 | | 1 | | 50 | .000 | |  |
| 3 | | .926^c^ | .858 | | .849 | | 1.35382 | .018 | | 6.250 | | 1 | | 49 | .016 | |  |
| ^a^ Predictors: (Constant), Salinity | | | | | | | | | | | | | | | | |  |
| ^b^ Predictors: (Constant), Salinity, pH | | | | | | | | | | | | | | | | |  |
| ^c^ Predictors: (Constant), Salinity, pH, Chl-a | | | | | | | | | | | | | | | | |  |
| ^d^ Dependent Variable: Condition index | | | | | | | | | | | | | | | | |  |
| Coefficients^a^ | | | | | | | | | | | | | | | | |  |
| Model | | | | Unstandardized Coefficients | | | | | Standardized Coefficients | | t | | Sig. | | |  |  |
|  |  |  |  | B | | Std. Error | | | Beta | |  |  |  |  |  |  |  |
| 1 | (Constant) | | | -1.269 | | .544 | | |  | | -2.334 | | .024 | | |  |  |
|  | Salinity | | | .293 | | .024 | | | .866 | | 12.361 | | .000 | | |  |  |
| 2 | (Constant) | | | -63.374 | | 11.704 | | |  | | -5.415 | | .000 | | |  |  |
|  | Salinity | | | .218 | | .024 | | | .645 | | 9.195 | | .000 | | |  |  |
|  | pH | | | 7.855 | | 1.479 | | | .373 | | 5.310 | | .000 | | |  |  |
| 3 | (Constant) | | | -47.088 | | 12.900 | | |  | | -3.650 | | .001 | | |  |  |
|  | Salinity | | | .213 | | .023 | | | .631 | | 9.416 | | .000 | | |  |  |
|  | pH | | | 5.695 | | 1.651 | | | .270 | | 3.448 | | .001 | | |  |  |
|  | Chl-a | | | .259 | | .104 | | | .175 | | 2.500 | | .016 | | |  |  |
| ^a^ Dependent Variable: Condition Index | | | | | | | | | | | | | | | | | |

**S2 Table. Summary of linear model results (continued)**

| Model, *y_h_* | | | | | | | | | |
| --- | --- | --- | --- | --- | --- | --- | --- | --- | --- |
| Model | R | R^2^ | Adjusted R^2^ | Std. Error of the Estimate | Change Statistics | | | | |
|  |  |  |  |  | R Square Change | F Change | df1 | df2 | Sig. F Change |
| 1 | .884^a^ | .782 | .778 | 1.17646 | .782 | 182.972 | 1 | 51 | .000 |
| 2 | .916^b^ | .839 | .833 | 1.02066 | .057 | 17.759 | 1 | 50 | .000 |
| 3 | .925^c^ | .855 | .846 | .97826 | .016 | 5.428 | 1 | 49 | .024 |
| ^a^ Predictors: (Constant), Salinity | | | | | | | | | |
| ^b^ Predictors: (Constant), Salinity, Chl-a | | | | | | | | | |
| ^c^ Predictors: (Constant), Salinity, Chl-a, DO | | | | | | | | | |
| ^d^ Dependent Variable: Average shell height | | | | | | | | | |

| **Coefficients^a^** | | | | | | | |
| --- | --- | --- | --- | --- | --- | --- | --- |
| Model | | Unstandardized Coefficients | | Standardized Coefficients | t | Sig. |  |
|  |  | B | Std. Error | Beta |  |  |  |
| 1 | (Constant) | -1.017 | .363 |  | -2.801 | .007 |  |
|  | Salinity | .214 | .016 | .884 | 13.527 | .000 |  |
| 2 | (Constant) | -1.878 | .375 |  | -5.002 | .000 |  |
|  | Salinity | .186 | .015 | .771 | 12.274 | .000 |  |
|  | Chl-a | .281 | .067 | .265 | 4.214 | .000 |  |
| 3 | (Constant) | -4.319 | 1.108 |  | -3.899 | .000 |  |
|  | Salinity | .161 | .018 | .665 | 8.827 | .000 |  |
|  | Chl-a | .220 | .069 | .207 | 3.184 | .003 |  |
|  | DO | .050 | .021 | .189 | 2.330 | .024 |  |
| ^a.^ Dependent Variable: Shell height | | | | | | | |
